# A serum metabolite-based machine learning model predicts response to neoadjuvant immunotherapy in mismatch repair-deficient colorectal cancer

**DOI:** 10.3389/fonc.2026.1730155

**Published:** 2026-02-19

**Authors:** Tao Ma, Weili Zhang, Yuxi Pan, Guojie Long, Xiuwei Mi, Junfeng Jiang, Fan Bai, Hao Zhang, Tuo Hu, Ziyang Zeng, Weidong Pan

**Affiliations:** 1 Department of Pancreatic Hepatobiliary Surgery, The Sixth Affiliated Hospital, Sun Yat-sen University, Guangzhou, China; 2 Biomedical Innovation Center, The Sixth Affiliated Hospital, Sun Yat-sen University, Guangzhou, China; 3 Department of Colorectal Surgery, Sun Yat-sen University Cancer Center, Guangzhou, Guangdong, China; 4 State Key Laboratory of Oncology in South China, Collaborative Innovation Center for Cancer Medicine, Sun Yat-sen University Cancer Center, Guangzhou, China; 5 Department of Colorectal Surgery, The Sixth Affiliated Hospital, Sun Yat-sen University, Guangzhou, China; 6 Guangdong Provincial Key Laboratory of Colorectal and Pelvic Floor Diseases, Guangdong Institute of Gastroenterology, The Sixth Affiliated Hospital, Sun Yat-sen University, Guangzhou, China

**Keywords:** colorectal cancer, immune checkpoint inhibitors, machine learning, predictive model, serum metabolomics

## Abstract

**Background:**

Colorectal cancer (CRC) with microsatellite instability-high (MSI-H) or mismatch repair-deficient (dMMR) shows significant sensitivity to immune checkpoint inhibitors (ICIs). However, a considerable proportion of patients still exhibit primary or acquired resistance to ICIs. Until now, efficient and non-invasive biomarkers for accurately predicting immunotherapy efficacy remain unavailable.

**Methods:**

In this multicenter study, we employed liquid chromatography–mass spectrometry (LC–MS) and enzyme-linked immunosorbent assay (ELISA) to identify and validate serum metabolites associated with response to immunotherapy. Using machine learning algorithms, we constructed a random forest predictive model based on a panel of five metabolites. This model, termed the 5-Metabolite Predictive Model (5-MPM), incorporates prostaglandin E2 (PGE2), tryptophan, arginine, citrulline, and histidine.

**Results:**

The 5-MPM model demonstrated robust predictive performance in both training cohort and external validation cohort, with AUC values of 0.85 and 0.88, respectively. The SHAP analysis elucidated the contribution of each metabolite to model predictions. Integrating above five metabolites with metastasis stage did not further improve the predictive performance of this model.

**Discussion:**

This study provides the first systematic characterization of metabolic reprogramming in dMMR colorectal cancer with different response to immunotherapy, and establishes a non-invasive, high-precision predictive tool that offers a new basis for individualized therapeutic decision-making.

## Introduction

MSI-H/dMMR CRC constitutes a distinct molecular subtype, accounting for 5%~15% of all cases (1, 2). This subtype is characterized by pronounced genomic instability, a high mutational burden, and distinctive immune features (3). These features render dMMR CRCs particularly sensitive to immune checkpoint blockade, with anti-PD-1/PD-L1 antibodies established as the standard first-line therapy for metastatic disease (4). However, about 20–40% of dMMR CRC patients exhibit primary resistance, and many develop acquired resistance after an initial response (5, 6). There is therefore a pressing need to develop more accurate approaches to predict immunotherapy efficacy.

Several biomarkers, including PD-L1 expression, tumor mutational burden (TMB) and tumor-infiltrating lymphocyte (TIL) density, have been associated with immunotherapy response (7). Advances in genomic, transcriptomic and single-cell sequencing technologies further facilitated biomarker discovery and development (8). However, most of these approaches rely on tumor tissue, which is often difficult to obtain, especially for patients who are not suitable for surgery. This underscores an increasing need for non-tissue-based biomarker strategies that combine high accuracy with minimal invasiveness. In this context, serum or plasma tests, which enable longitudinal, noninvasive monitoring, represent a promising approach for predicting immunotherapy efficacy (9).

Tumor genetic heterogeneity, together with its interactions with the immune microenvironment, collectively shapes metabolic reprogramming (10). Several metabolites, including lactate (11), kynurenine (12), and prostaglandin E2 (PGE_2_) (13), have been shown to directly modulate response to ICIs. Serum metabolomics enables systematic profiling of metabolic alterations in cancer patients, and its non-invasive nature enhances clinical applicability. By simultaneously detecting hundreds of metabolites, serum metabolomics generates highly complex datasets. However, challenges such as limited clinical sample sizes and the complexity of nonlinear modeling underscore the need for machine learning approaches.

In this study, 151 dMMR CRC patients with neoadjuvant immunotherapy were enrolled across two independent centers. Employing liquid chromatography–mass spectrometry (LC–MS)-based targeted metabolomics, we quantitatively profiled serum samples from 30 pair-matched responders and non-responders to immunotherapy. After identifying differentially expressed metabolites and confirming their significance across the entire cohort, a machine learning-based predictive model was developed and independently validated. Through comprehensive performance evaluation, including accuracy, receiver operating characteristic (ROC) analysis, and positive predictive value, we established a robust predictive framework to identify patient subgroups most likely to benefit from ICIs therapy.

## Methods

### Study design and patient cohort

This study included two cohorts comprising 151 dMMR CRC patients who received neoadjuvant anti-PD-1/PD-L1 immunotherapy. The training cohort (SYSU-SAH, n=121) was derived from the Sixth Affiliated Hospital of Sun Yat-sen University (SYSU-SAH), with serum samples collected between January 2019 and January 2024 for model development. The external validation cohort (SYSUCC, n=30) was obtained from the Cancer Center of Sun Yat-sen University (SYSUCC), with samples collected from January 2023 to January 2024 for model performance evaluation. This study was approved by the ethics committees of both participating institutions: the Sixth Affiliated Hospital of Sun Yat-sen University (Approval No. 2025ZSLYEC-397) and Sun Yat-sen University Cancer Center (Approval No. B2023-188-04). Immunotherapy efficacy was evaluated by postoperative pathological assessment. Patients were classified as responders or non-responders based on major pathological response (MPR, defined as ≤10% residual viable tumor cells) or pathological complete response (pCR, defined as no residual viable tumor cells). Clinical data, including sex, age, tumor stage, smoking history, tumor location, tumor differentiation, and mismatch repair protein expression, were obtained from medical records. Missing clinical data were addressed using multiple imputation methods.

### Metabolomics analysis and biomarker identification

Serum samples from 15 immunotherapy responders and 15 non-responders in the training cohort were processed for targeted metabolomics analysis to identify potential metabolic biomarkers. Differential metabolites were screened based on the following criteria: Fold change (FC) > 1.5 or < 0.67 and False discovery rate (FDR) < 0.05, which identified nine candidate metabolites associated with immunotherapy response. Subsequently, the concentrations of these nine metabolites were quantified in serum samples. To avoid data leakage and ensure rigorous validation, feature selection was performed exclusively using the training cohort (SYSU-SAH, n=121). Five of these differential metabolites demonstrated significantly statistical difference in the training cohort and were selected as features for model development.

### Machine learning model development

Two sets of predictive models were developed. The first set utilized the concentrations of five differentially expressed metabolites (PGE_2_, tryptophan, arginine, citrulline, and histidine) as input features. The second set combined these five metabolites with clinical variables, including sex, age, tumor stage, smoking history, tumor location, tumor differentiation, and mismatch repair protein expression, as input features. To assess for multicollinearity among the five metabolite features, we calculated the Variance Inflation Factor (VIF). The VIF values for PGE2 (1.13), Tryptophan (1.08), Histidine (1.08), Arginine (1.16), and Citrulline (1.04) were all well below the common threshold of 5, indicating that multicollinearity was not a significant concern for our model development. Both sets of models were constructed using six machine learning algorithms: logistic regression (LR), k-nearest neighbors (KNN), random forest (RF), support vector machine (SVM), linear discriminant analysis (LDA), and extreme gradient boosting (XGBoost). The models were trained in the training cohort (n = 121) with 5-fold cross-validation (repeated five times) to optimize performance and mitigate overfitting. Hyperparameters for each algorithm were optimized using grid search to maximize the area under the receiver operating characteristic curve (ROC-AUC). Feature selection was conducted using recursive feature elimination (RFE) combined with the random forest algorithm to identify the most predictive features. The SHAP (SHapley Additive exPlanations) method was employed to evaluate feature importance, with SHAP values calculated for each feature based on the RF model. All models were implemented in the R environment using the “tidymodels” and “SHAP” (version 0.42.0) packages. Data preprocessing involved standard deviation normalization (StandardScaler) and multiple imputation for missing values (missing rate < 5%).

### Model evaluation

Model performance was assessed using multiple metrics, including ROC-AUC, accuracy, F1 score, Matthews correlation coefficient (MCC), negative predictive value (NPV), positive predictive value (PPV), recall, sensitivity, and specificity. These metrics were calculated separately in the training cohort (n = 121) using 5-fold cross-validation and in the external validation cohort (n = 30) to evaluate robustness and generalizability of the model. The predictive performance of each model was compared to identify the optimal algorithm for predicting neoadjuvant immunotherapy efficacy.

### Statistical analyses

Statistical analyses were conducted using R (version 4.5.0). Continuous variables were compared using Student’s t-tests or Wilcoxon rank-sum tests, depending on data distribution. Categorical variables were analyzed with chi-square tests. The statistical comparison of the Receiver Operating Characteristic (ROC) curves between the 5-MPM model (containing only five metabolites) and the combined model (integrating the five metabolites with M stage) was performed using DeLong’s test. A p-value < 0.05 was considered statistically significant. Machine learning analyses were performed using the tidymodels package, with standardized data preprocessing steps, including normalization via StandardScaler and multiple imputation for missing values (missing rate < 5%).

## Results

### The overall workflow of study design, patient enrollment and data collection

**Figure 1:**
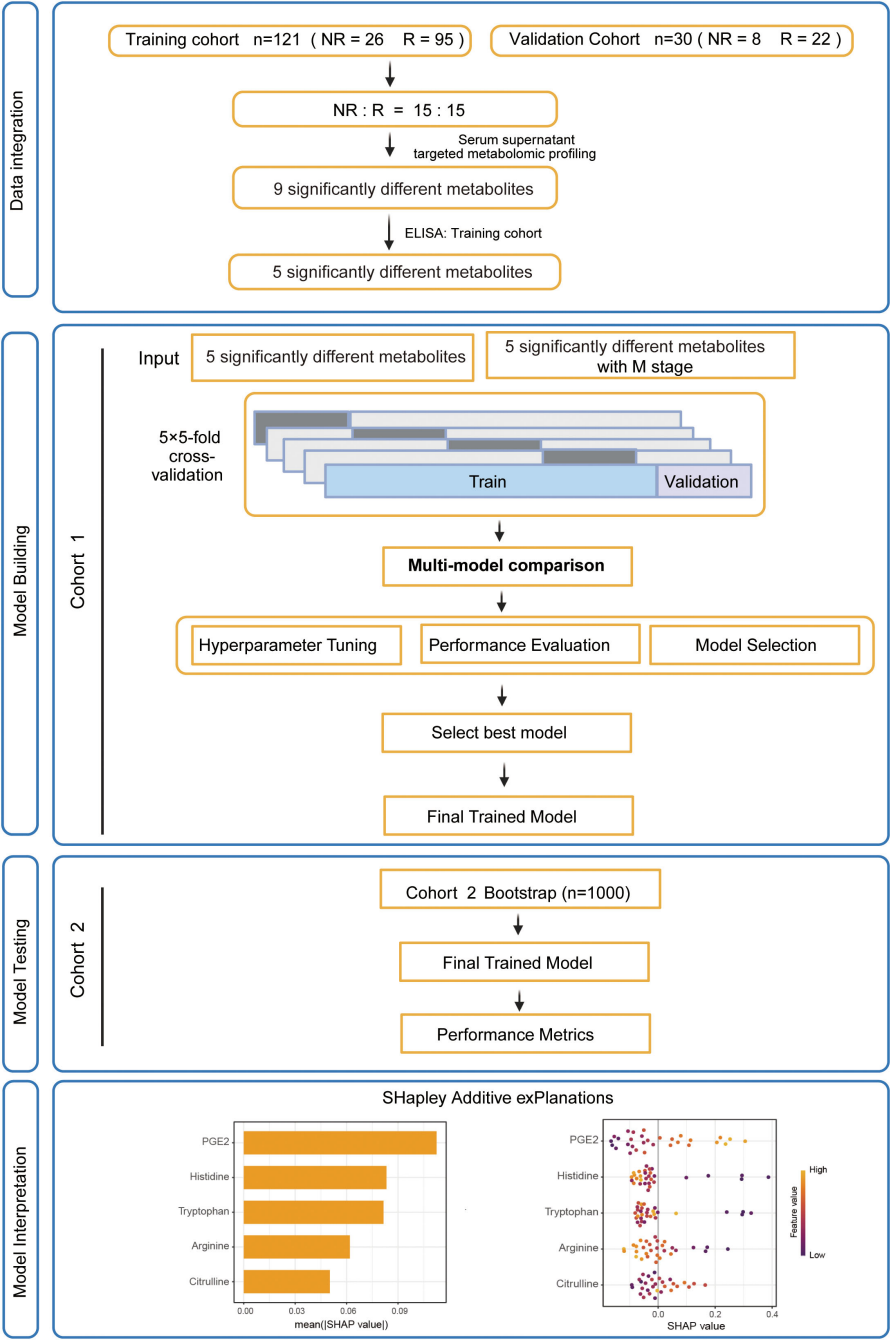
Overall workflow of patients enrollment, sample processing, and model construction. The diagram illustrates the overall study design, including patient enrollment, serum metabolomic profiling, identification and validation of differential metabolites, machine learning–based model development, external validation, and SHAP-based interpretation.

Serum samples from 151 dMMR/MSI-H CRC patients with neoadjuvant anti-PD1/PD-L1 immunotherapy were analyzed, their clinical characteristics were provided in **Table 1**. In SYSUSAH cohort (n = 121), 95 patients were classified as responders and 26 as non-responders, whereas SYSUCC cohort (n = 30) consisted of 22 responders and 8 non-responders. Serum samples from 15 immunotherapy responders and 15 immunotherapy non-responders in SYSUSAH cohort were processed for targeted metabolomic, which revealed a panel of candidate metabolites distinguishing responders from non-responders. These metabolites were subsequently validated in the entire study population, and multiple predictive models were developed based on the validated biomarkers. The optimal model demonstrated robust performance and generalizability. The overall workflow of patient enrollment, metabolite screening, model construction, validation, and interpretation was presented in **Figure 1**.

**Table 1:** Comparison of the baseline characteristics in patients.

Overall,N = 151				Cohort 1, N = 121			Cohort 2, N = 30		
Characteristic	NR (N=34) No. (%)	R (N=117) No. (%)	p-value	NR (N=26) No. (%)	R (N=95) No. (%)	p-value	NR (N=8) No. (%)	NR (N=8) No. (%)	p-value
SEX, n (%)			0.42			0.82			0.38
Female	20 (59%)	78 (67%)		16 (62%)	62 (65%)		4 (50%)	16 (73%)	
Male	14 (41%)	39 (33%)		10 (38%)	33 (35%)		4 (50%)	6 (27%)	
AGE, Mean (SD)	51.88 (13.83)	50.08 (15.20)	0.53	53.62 (14.48)	49.74 (15.54)	0.23	46.25 (10.28)	51.55 (13.86)	0.32
Clinical T stage, n (%)			0.49			>0.99			0.16
T3	6 (18%)	28 (24%)		6 (23%)	22 (23%)		0 (0%)	6 (27%)	
T4	28 (82%)	89 (76%)		20 (77%)	73 (77%)		8 (100%)	16 (73%)	
Clinical N stage, n (%)			0.96			0.72			>0.99
N0	4 (12%)	16 (14%)		1 (3.8%)	8 (8.4%)		3 (38%)	8 (36%)	
N1	11 (32%)	40 (34%)		6 (23%)	27 (28%)		5 (63%)	13 (59%)	
N2	19 (56%)	61 (52%)		19 (73%)	60 (63%)		0 (0%)	1 (4.5%)	
pM, n (%)			0.005			0.029			0.06
0	17 (50%)	90 (77%)		13 (50%)	71 (75%)		4 (50%)	19 (86%)	
1	17 (50%)	27 (23%)		13 (50%)	24 (25%)		4 (50%)	3 (14%)	
MSH2, n (%)			0.22			0.13			0.67
0	15 (44%)	37 (32%)		10 (38%)	21 (22%)		5 (63%)	16 (73%)	
1	19 (56%)	80 (68%)		16 (62%)	74 (78%)		3 (38%)	6 (27%)	
MSH6, n (%)			0.68			>0.99			0.2
0	10 (29%)	41 (35%)		6 (23%)	24 (25%)		4 (50%)	17 (77%)	
1	24 (71%)	76 (65%)		20 (77%)	71 (75%)		4 (50%)	5 (23%)	
MLH1, n (%)			0.7			0.66			>0.99
0	15 (44%)	57 (49%)		10 (38%)	42 (44%)		5 (63%)	15 (68%)	
1	19 (56%)	60 (51%)		16 (62%)	53 (56%)		3 (38%)	7 (32%)	
PMS2, n (%)			0.56			0.82			0.68
0	17 (50%)	67 (57%)		14 (54%)	55 (58%)		3 (38%)	12 (55%)	
1	17 (50%)	50 (43%)		12 (46%)	40 (42%)		5 (63%)	10 (45%)	
Primary tumour location, n (%)			0.18			0.26			0.21
Ascending colon	5 (15%)	26 (22%)		3 (12%)	23 (24%)		2 (25%)	3 (14%)	
Transverse colon	7 (21%)	17 (15%)		7 (27%)	13 (14%)		0 (0%)	4 (18%)	
Descending colon	7 (21%)	12 (10%)		3 (12%)	10 (11%)		4 (50%)	2 (9.1%)	
Hepatic flexure		12 (10%)		0 (0%)	10 (11%)		0 (0%)	2 (9.1%)	
Splenic flexure	0 (0%)	4 (3.4%)		0 (0%)	2 (2.1%)		0 (0%)	2 (9.1%)	
Sigmoid colon	4 (12%)	17 (15%)		3 (12%)	10 (11%)		1 (13%)	7 (32%)	
Rectum	11 (32%)	29 (25%)		10 (38%)	27 (28%)		1 (13%)	2 (9.1%)	
Histological appearance, n (%)			0.13			0.17			0.53
Poorly differentiated	9 (26%)	16 (14%)		6 (23%)	12 (13%)		3 (38%)	4 (18%)	
Moderately differentiated	21 (62%)	92 (79%)		16 (62%)	75 (79%)		5 (63%)	17 (77%)	
Well differentiated	4 (12%)	9 (7.7%)		4 (15%)	8 (8.4%)		0 (0%)	1 (4.5%)	
Smoking, n (%)			>0.99			0.75			0.42
0	28 (82%)	95 (81%)		22 (85%)	83 (87%)		6 (75%)	12 (55%)	
1	6 (18%)	22 (19%)		4 (15%)	12 (13%)		2 (25%)	10 (45%)	

### Screening and validation of metabolites associated with immunotherapy response

**Figure 2:**
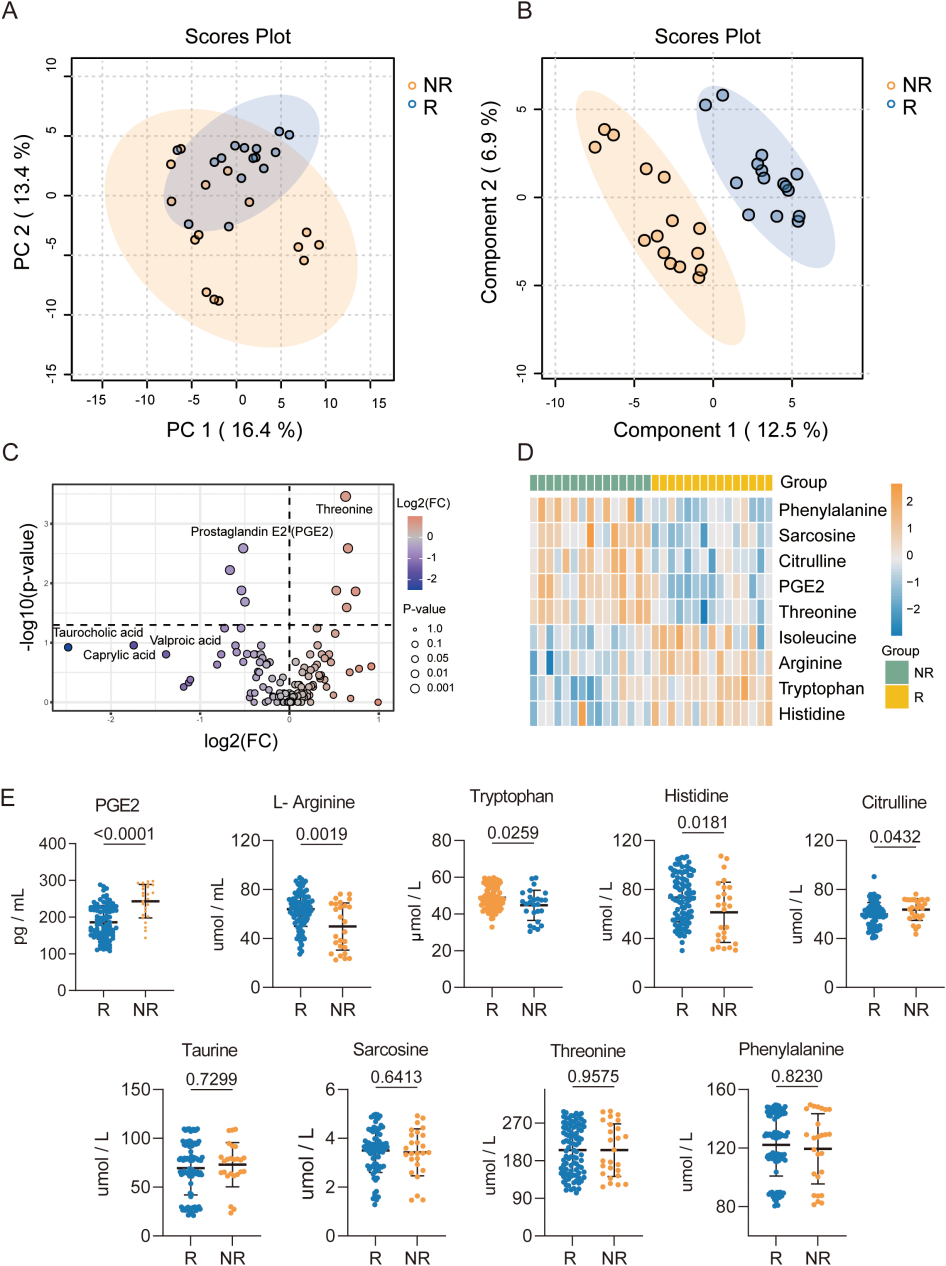
Identification and validation of differential serum metabolites associated with immunotherapy response. **(A)**. Principal component analysis (PCA) score plot showing the overall separation between immunotherapy responders and non-responders based on targeted metabolomics profiles. **(B)**. Partial least squares-discriminant analysis (PLS-DA) score plot demonstrating the distinct clustering between immunotherapy responders and non-responders. **(C)**. Volcano plot displaying the distribution of differential metabolites, with significantly upregulated and downregulated metabolites highlighted (Fold change >1.5 or Fold change < -0.67 and FDR < 0.05). **(D)**. Heatmap of significantly altered metabolites, illustrating distinct metabolic patterns between immunotherapy responders and non-responders. **(E)**. ELISA validation of nine representative differential metabolites in serum samples from training Cohort (n=121). Among them, five metabolites showing significantly statistical differences between immunotherapy responders and non-responders (*p* < 0.05).

To identify dysregulated metabolites associated with immunotherapy response in dMMR/MSI-H CRC patients, serum samples from 15 non-responders (NR) and 15 responders (R) in SYSUSAH cohort were processed for targeted metabolomic analysis. Principal component analysis (PCA) and orthogonal projections to latent structures–discriminant analysis (OPLS-DA) revealed distinct serum metabolic profiles between responders and non-responders (**Figures 2A, B**). Compared with responders, nine differential metabolites were identified in serum from the non-responders (FDR < 0.05; Fold change > 1.5 or < 0.67) (**Figure 2C**). These dysregulated metabolites included seven amino acids (threonine, isoleucine, histidine, tryptophan, citrulline, phenylalanine, arginine), an inflammatory mediator prostaglandin E2 (PGE2), and a methylation-related metabolite sarcosine (**Figure 2C**). Unsupervised clustering analysis showed that these nine metabolites could clearly classified the 30 samples into NR and R groups (**Figure 2D**). These differential metabolites levels were subsequently validated with enzyme-linked immunosorbent assay (ELISA) in training Cohort (SYSU-SAH, n = 121). This validation results revealed that five metabolites (PGE2, arginine, citrulline, histidine, and tryptophan) were consistently significantly different between R and NR groups (*P* < 0.05, **Figure 2E**).

### The metabolite-based model for predicting immunotherapy response

**Figure 3:**
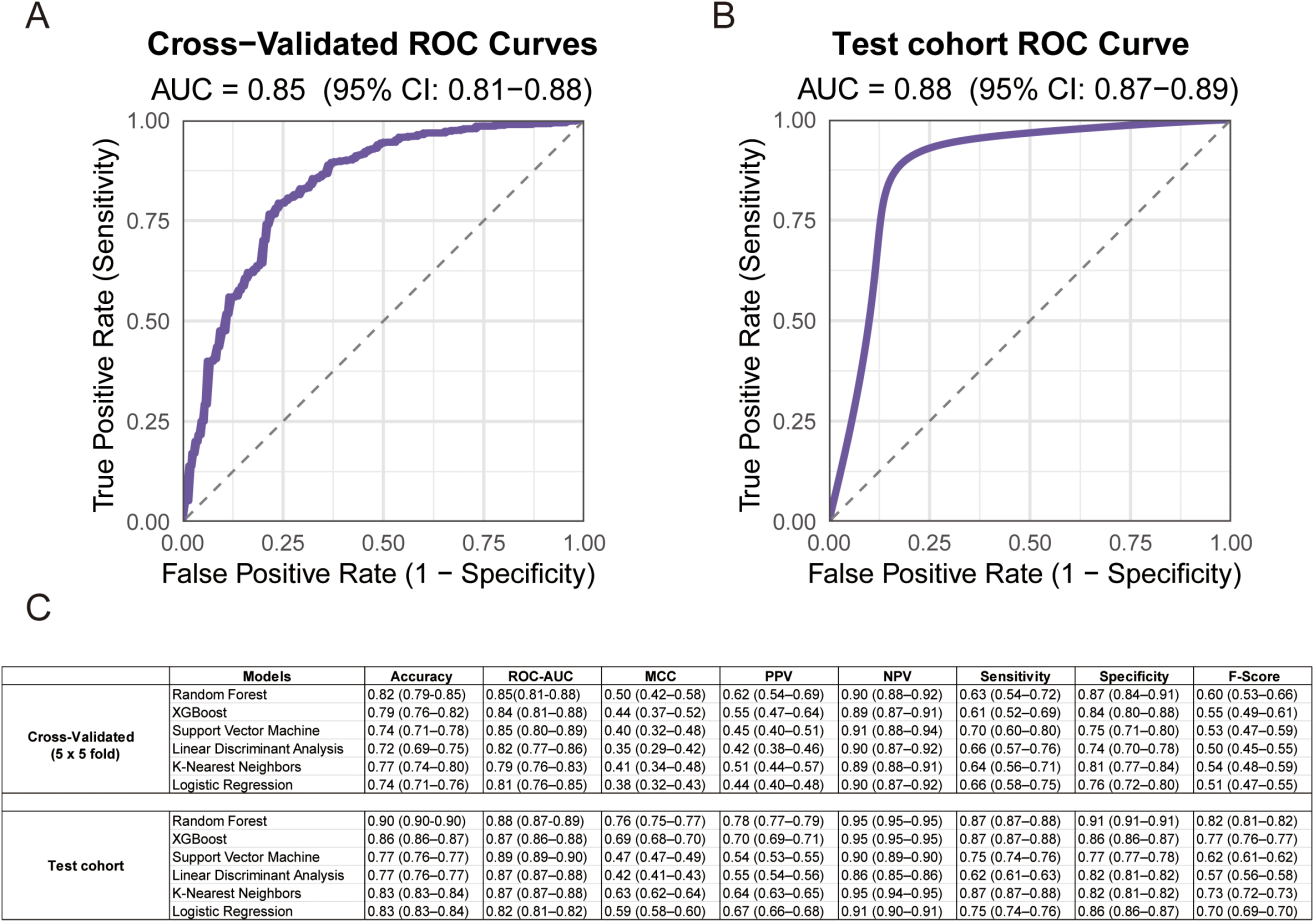
Performance of the 5-metabolite predictive model across the training and validation cohorts. **(A)**. ROC curves of the 5-MPM model constructed with random forest algorithm, which is obtained from 5-fold cross-validation repeated five times in the training cohort. **(B)**. ROC curve of the 5-MPM model in the training cohort, with 95% confidence interval **(CI)** estimated by 1000 bootstrap resamples. **(C)**. Comparison across six models, 5-MPM (random forest), XGBoost, support vector machine, logistic regression (LR), Linear Discriminant Analysis, K-Nearest Neighbors, Logistic Regression and support vector machine (SVM). Data are shown by mean area under the ROC curve (AUC), sensitivity, specificity, and accuracy derived from 5-fold cross-validation repeated five times in the training and testing cohort, as estimated with 95% CIs computed by 1000-times bootstrap resampling.

Based on above five differential metabolites (PGE2, arginine, citrulline, histidine, and tryptophan), predictive models for immunotherapy efficacy were developed with six machine learning algorithms applied to the SYSUSAH (training) cohort (n = 121). Upon evaluation with five-fold cross-validation repeated five times, the RF model demonstrated the best overall performance, achieving an AUC of 0.85 (95% CI: 0.81–0.88) and an MCC of 0.50 (95% CI: 0.42–0.58), thereby demonstrating robust stability in handling class-imbalanced data. In an independent testing cohort (n = 30), the RF model exhibited strong generalization capability, as evidenced by 1000 bootstrap resampling validations: AUC increased to 0.88 (95% CI: 0.87–0.89) and MCC reached 0.76 (95% CI: 0.75–0.77) (**Figures 3A, B**). Although the Support Vector Machine model achieved a slightly higher AUC (reported as 0.89; 95% CI: 0.89–0.90), its performance on other metrics—including precision (0.77, 95% CI: 0.76–0.77), MCC (0.48, 95% CI: 0.47–0.49), sensitivity (0.75, 95% CI: 0.74–0.76), specificity (0.77, 95% CI: 0.77–0.78), and F1-score (0.62, 95% CI: 0.61–0.63)—was inferior to the RF model (**Figure 3C**), indicating that RF provided more balanced and comprehensive predictive performance. In summary, the metabolite-based 5-MPM model developed with Random Forest demonstrated excellent and well-balanced performance in both cross-validation and independent testing, underscoring its potential application in predicting immunotherapy efficacy.

### The predictive model for immunotherapy response based on metabolites and M stage

**Figure 4:**
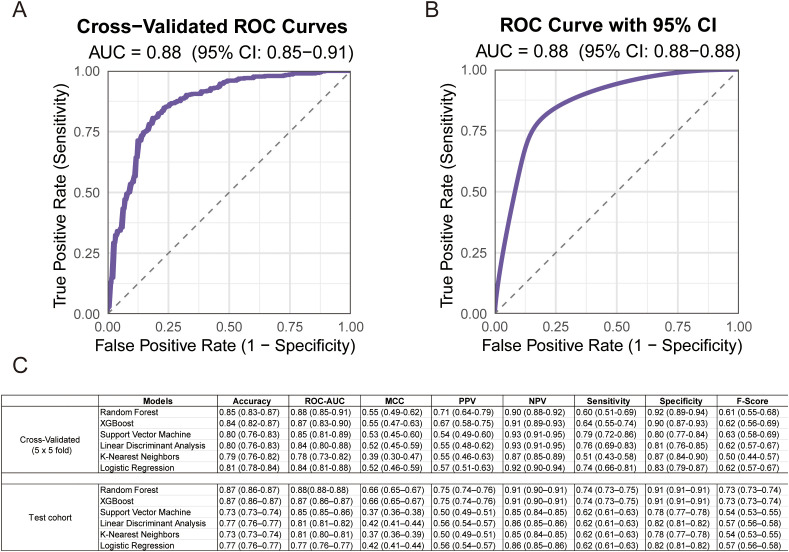
Performance of the integrated model combining 5-MPM with clinical M stage. **(A)**. ROC curves of the 5-MPM+M stage model constructed with random forest algorithm, which is obtained from 5-fold cross-validation repeated five times in the training cohort. **(B)**. ROC curve of the 5-MPM+M stage model in the training cohort, with 95% CI estimated by 1000 bootstrap resamples. **(C)**. Comparison across six models, 5-MPM+M stage (random forest), logistic regression (LR), support vector machine (SVM), extreme gradient boosting (XGBoost), k-nearest neighbors (KNN), and multilayer perceptron (MLP). Data are shown by mean area under the ROC curve (AUC), sensitivity, specificity, and accuracy derived from 5-fold cross-validation repeated five times in the training and testing cohort, as estimated with 95% CIs computed by 1000-times bootstrap resampling.

To further enhance predictive performance, we combined above five metabolite biomarkers (PGE2, arginine, citrulline, histidine, and tryptophan) with M stage, which was associated with immunotherapy efficacy (*p* < 0.05) in our cohort. Using the training set from SYSUSAH cohort (n = 121), a combined predictive model was constructed and evaluated with five-fold cross-validation repeated five times. The RF integrated model demonstrated superior overall performance during cross-validation, with an accuracy of 0.849 (95% CI: 0.830–0.869) and AUC of 0.88 (95% CI: 0.85–0.91) (**Figures 4A, C**). For external validation, we tested this model in another independent dMMR/MSI-H cohort from external validation cohort (SYSUCC, n = 30) using 1000 bootstrap resamples. The RF integrated model achieved an accuracy of 0.87 (95% CI: 0.86–0.87) and AUC of 0.88 (95% CI: 0.88–0.88) (**Figures 4B, C**). Overall, although the RF-integrated model (five metabolites and M stage) achieved the best performance, its improvement over the metabolites-based 5-MPM model was not statistically significant (P > 0.05).

### The interpretation of metabolites-based 5-MPM model via SHAP

**Figure 5:**
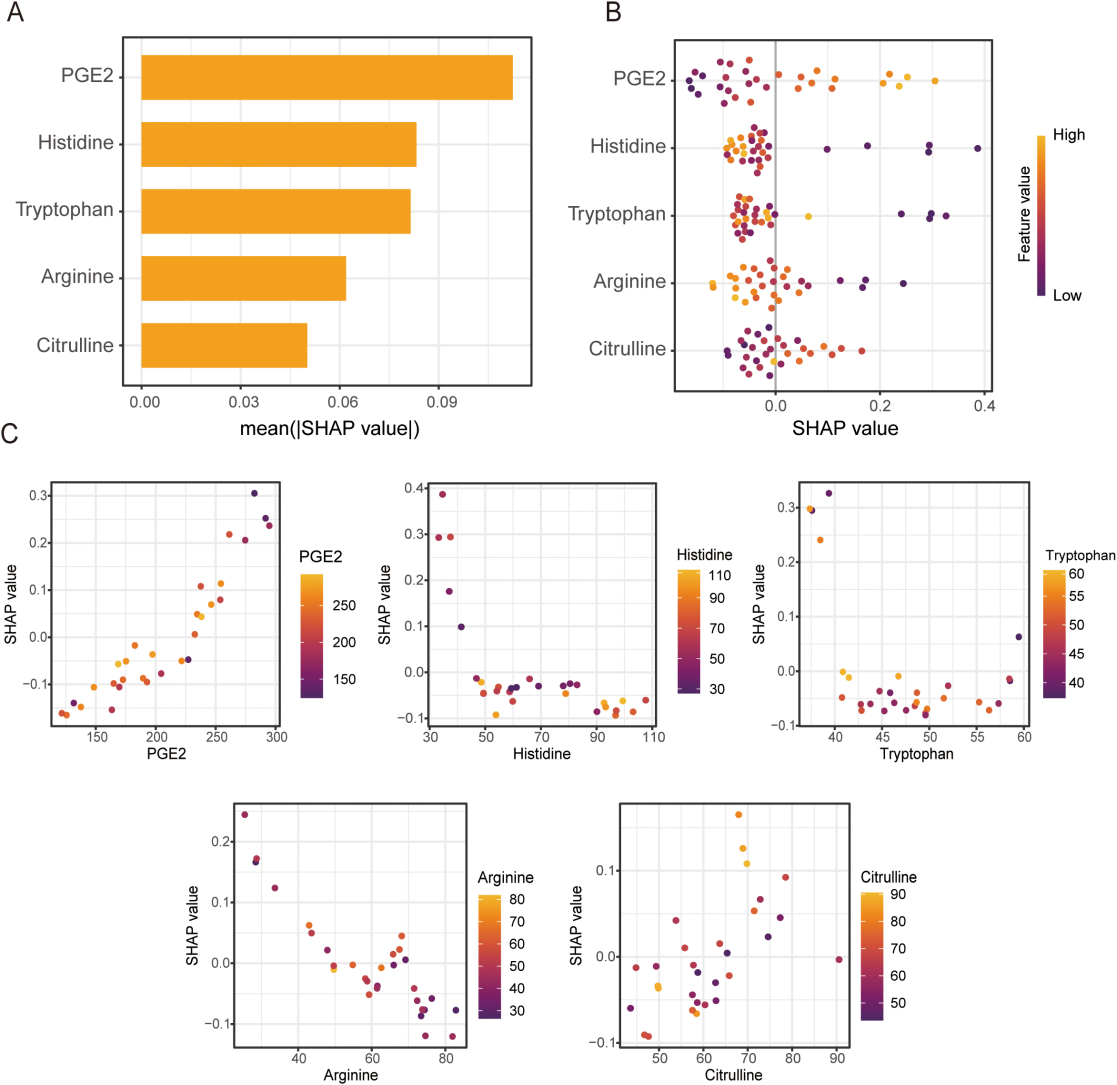
SHAP-based interpretation of metabolic contributions in the 5-MPM model. **(A)**. Bar plot of mean absolute SHAP values ranking the importance of each metabolite in the 5-MPM model, indicating which metabolites contributed most to predictive performance. **(B)**. Summary plot of SHAP values showing overall contribution of each metabolite to predictive performance of the 5-MPM model. Each point represents a sample, with color indicating relative value of each feature (red = high, blue = low), and horizontal axis representing the SHAP value (impact on model output). **(C)**. SHAP dependence plots illustrating relationship between indicated metabolite levels and their corresponding SHAP values in the 5-MPM model, illustrating association between metabolite concentration and prediction direction or magnitude.

To interpret the metabolites-based 5-MPM model, SHAP method was applied to quantify the contribution of each metabolite to its predictive performance. As shown in the SHAP summary bar plot (**Figure 5A**), mean absolute SHAP values ranked metabolites by their importance in 5-MPM model in descending order: PGE_2_, histidine, tryptophan, arginine, and citrulline. Furthermore, the SHAP summary bee swarm plot depicts the direction and magnitude of each metabolite’s impact on the model output. Elevated levels of PGE_2_ and citrulline were associated with higher SHAP values, whereas lower levels of histidine, tryptophan, and arginine contributed to better prediction scores (**Figure 5B**). Moreover, the SHAP dependence plots further elucidated how individual metabolites influenced the model output. As shown in **Figure 5C**, a SHAP value greater than zero indicated a positive contribution toward predicting non-response (NR), corresponding to an increased risk of poor immunotherapy outcome. Thus, higher levels of PGE_2_ and citrulline, together with lower levels of tryptophan, arginine, and histidine, were associated with increased SHAP values, suggesting a greater likelihood of inferior immunotherapy efficacy.

### Verification of the necessity of the selected metabolites via ablation study

To verify the necessity of the selected metabolite panel, we performed a “leave-one-out” ablation study. Specifically, we trained five derivative models, each excluding exactly one metabolite, while maintaining identical hyperparameter settings and training/validation splits as the original 5-MPM model. Our analysis revealed that the full 5-MPM model (Accuracy = 0.90, AUC = 0.88) consistently outperformed all reduced models. As shown in **Supplementary Table S1**, the exclusion of any single metabolite resulted in a decrease of predictive performance (Accuracy range: 0.63–0.87; AUC range: 0.68–0.88). These findings confirm that each metabolite provides unique and essential information to the 5-MPM model.

### Decision curve analysis for the predictive power of the 5-MPM model

**Figure 6:**
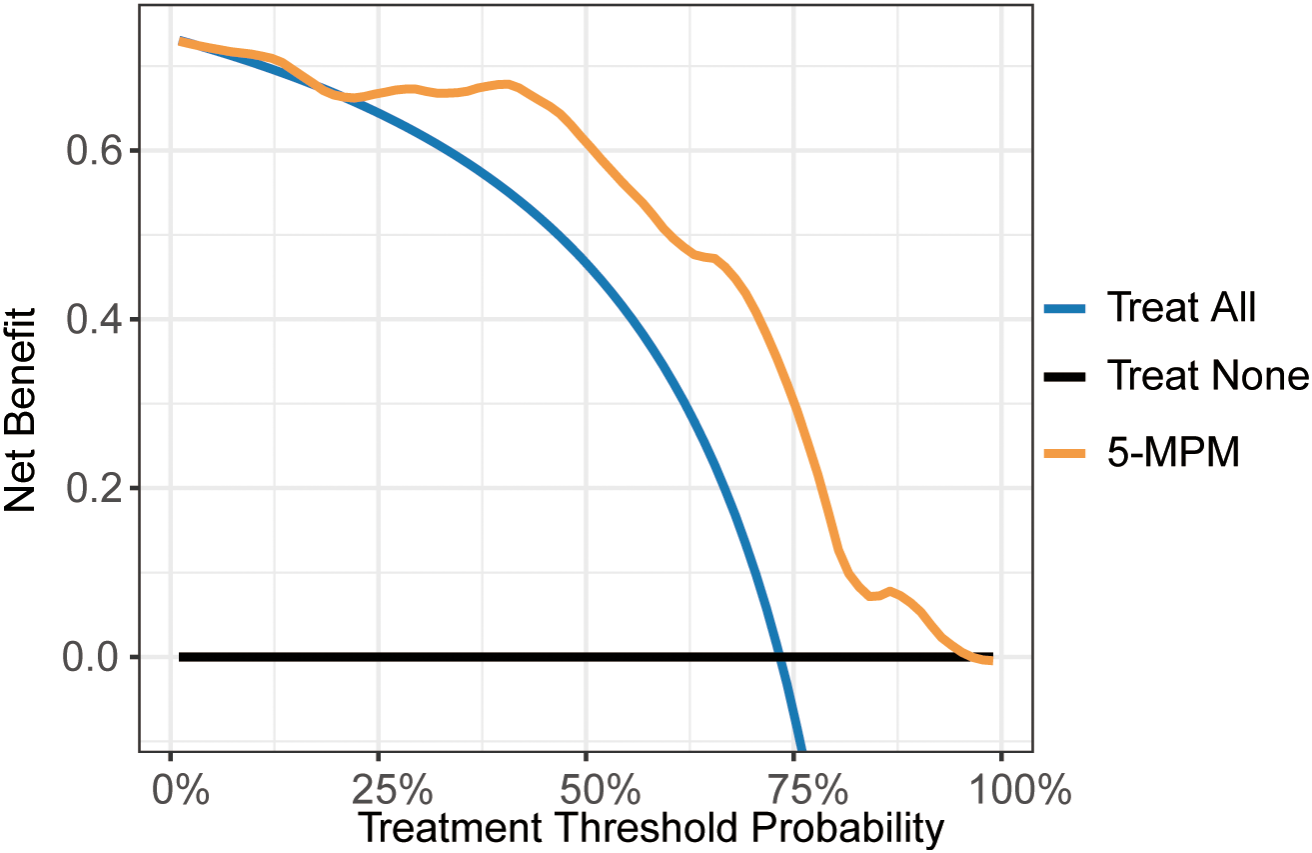
Decision curve analysis demonstrating the clinical application of the 5-MPM model. Decision curve analysis was performed to evaluate the clinical utility of the 5-MPM model by quantifying the net benefit across a range of threshold probabilities.

To evaluate the clinical utility of the 5-MPM model, the decision curve analysis (DCA) was performed across the training and external validation cohorts. As illustrated in **Figure 6**, the DCA curves demonstrate that the 5-MPM model yielded a higher net benefit for predicting immunotherapy response across a broad range of threshold probabilities compared with the ‘treat-all’ or ‘treat-none’ strategies. These findings suggest that the 5-MPM model could serve as a valuable tool for guiding clinical decision-making in neoadjuvant immunotherapy for dMMR/MSI-H CRC patients.

## Discussion

In this multicenter clinical study, we characterized serum metabolomic differences between responders and non-responders with dMMR CRC receiving neoadjuvant immunotherapy, which identified circulating metabolic biomarkers with potential predictive value. By integrating serum metabolomic data with machine learning algorithms, we developed a random forest–based predictive model, the 5-Metabolite Predictive Model (5-MPM), which incorporates PGE_2_, tryptophan, arginine, citrulline, and histidine. This model is intended to predict the therapeutic efficacy of neoadjuvant immunotherapy in dMMR CRC patients. In an independent validation cohort, the 5-MPM demonstrated robust discriminative performance, achieving an area under the curve (AUC) of 0.88 and a Matthews correlation coefficient (MCC) of 0.76. This model offers a non-invasive approach that has the potential for dynamic monitoring, with the potential to predict ICIs response. Furthermore, SHAP analysis was applied to enhance model interpretability, thereby offering a theoretical basis and practical guidance for timely adjustment of treatment strategies.

CRC is one of the most common and lethal malignancies worldwide (14). Immunotherapy has brought new hope to a subset of CRC patients, particularly those with MSI-H or dMMR tumors, in whom it demonstrates remarkable efficacy (15). However, primary resistance still occurs in approximately 20–40% of these patients (5). The lack of accurate predictive biomarkers hampers the early identification of non-responders, resulting in missed therapeutic opportunities and delayed interventions. Although several biomarkers, such as PD-L1 expression, TMB, and TILs, have been proposed, their clinical utility remains limited owing to their invasive assessment and suboptimal predictive performance (7). Considering the intimate crosstalk between cellular metabolism and the tumor immune microenvironment (16), our study investigates the potential of metabolites as predictors of immunotherapy outcomes. The metabolic biomarkers identified in this study exhibit strong predictive power, and a rigorously validated prediction model was constructed using machine learning algorithms. Notably, the five metabolites ultimately identified, PGE_2_, tryptophan, arginine, citrulline, and histidine, are not merely isolated entities, but rather integral participants in metabolic reprogramming and immunomodulatory processes within the tumor immune microenvironment. For example, arginine is converted into citrulline and nitric oxide via nitric oxide synthases to sustain T-cell activity, while its catabolism through arginase promotes the production of prostaglandin E_2_ (PGE_2_) (17). As for tryptophan, arginine, and histidine, they are functionally integrated through shared intermediates in the TCA cycle and cofactor- or methylation-dependent processes that sustain nucleotide biosynthesis, energy metabolism, and cellular signaling (18). Given the potent immunoregulatory effect of these metabolites, they form a tightly interconnected axis that collectively shapes the tumor immune microenvironment. This provides a strong biological rationale for their predictive capacity.

To further interpret the predictive contributions of key metabolites in the model, SHAP analysis was applied. The results indicated that elevated levels of PGE_2_ and citrulline are risk factors for poor response to immunotherapy. PGE_2_, an eicosanoid derived from arachidonic acid metabolism, is synthesized by cyclooxygenases (COX-1/2) and prostaglandin E synthase (PGES) (19). Studies have shown that PGE_2_ binds to EP2 and EP4 receptors to inhibit expression of IL-2 receptor γ-chain (IL-2Rγc) on T cells, thereby preventing the assembly of functional IL-2 receptor complexes. This suppression of IL-2 signaling directly impairs T cell proliferation and clonal expansion (13). Furthermore, PGE_2_ restricts the differentiation of TCF1^+^ stem-like CD8^+^ effector T cells from tumor-infiltrating lymphocytes (TILs), thereby promoting cancer immune evasion (20). PGE_2_ also enhances the differentiation and immunosuppressive function of regulatory T cells (Tregs), which suppresses IFN-γ and TNF production in NK cells (21), promotes M2 macrophage polarization and facilitates expansion and immunosuppressive activity of myeloid-derived suppressor cells (22). The role of citrulline in the tumor immune microenvironment appears more complex. Elevated L-citrulline has been associated with poor prognosis in triple-negative breast cancer (23). ASS1-mediated citrulline depletion is required for pro-inflammatory activation of macrophages, excess citrulline may suppress inflammatory responses by binding to JAK2 and inhibiting the JAK2–STAT1 signaling pathway (24). Conversely, citrulline also serves as a precursor for arginine regeneration to support T cell function and enhance antitumor response (25), suggesting a dual role of citrulline in regulating antitumor immunity.

In contrast, reduced levels of histidine, tryptophan, and arginine were associated with poor response to immunotherapy. Histidine, an essential amino acid and key substrate for protein synthesis, is critical for T cell activation, proliferation, and effector function (26). High plasma histidine levels were associated with improved overall survival in non-small cell lung cancer patients treated with anti-PD-1 (27). Dietary histidine suppresses pro-inflammatory cytokine released from macrophages (28), its downstream metabolite, histamine, exerts pleiotropic immunomodulatory effects, including inhibition of Treg activity, attenuation of MDSC-mediated immunosuppression, promotion of dendritic cell maturation and antigen presentation. Tryptophan, another essential amino acid, could enhance ICIs efficacy when supplemented orally, and elevated plasma tryptophan levels are associated with improved treatment responses (29). Arginine plays a central role in antitumor immunity by promoting T cell proliferation and activation, maintaining metabolic fitness, and supporting memory T cell formation (23, 30). Its availability directly regulates NK cell cytotoxicity, IFN-γ secretion, and *in vivo* persistence (31). Clinically, plasma arginine concentration is positively correlated with immunotherapy efficacy and patient survival, supporting its predictive value (32). In summary, the metabolites identified in this study not only demonstrate robust predictive performance in our model but also occupy central roles in the metabolic and immunoregulatory networks of the tumor immune microenvironment, providing a solid mechanistic rationale for their predictive power.

This study uncovered distinct patterns of metabolic reprogramming between dMMR CRC patients with divergent responses to immunotherapy, providing valuable data and novel biological insights to the field. Furthermore, by integrating machine learning with metabolomic profiling, we developed a highly accurate predictive model with strong clinical translation potential. This strategy aligns well with the paradigm of precision medicine, and the streamlined metabolite panel ensures reproducibility and simplicity, thereby supporting broad clinical application. In the future, evaluating relative rather than absolute metabolite levels may overcome limitations arising from platform variations and technical protocols, broadening the model’s applicability and advancing individualized immunotherapy.

This study has several limitations, including a modest cohort size (n = 151) and restriction to a Chinese population, which may limit the generalizability of findings. Although robust validation strategies were employed, including repeated cross-validation and resampling in the test set, further validation in larger, multicenter, and ethnically diverse cohorts is required to establish broader applicability. Notably, integrating metabolic features with clinical characteristics such as M stage did not further improve our model performance, suggesting that the 5-MPM model alone possesses strong predictive power. Further studies are needed to determine the optimal integration of this model into clinical decision pathways, including its use alongside or as an alternative to established biomarkers such as TMB or PD-L1.

## Conclusions

In summary, this study systematically characterizes the metabolic reprogramming patterns in dMMR CRC during immunotherapy and establishes a machine learning–based predictive model that accurately distinguishes responders from non-responders. Our findings deepen the understanding of metabolic regulation underlying immunotherapy response in CRC and highlight the value of machine learning–integrated multi-omics analysis for predicting immunotherapy outcomes and guiding clinical decisions.

## Data Availability

The datasets presented in this study can be found in online repositories. The names of the repository/repositories and accession number(s) can be found in the article/**Supplementary Material**.
